# Pairwise gene GO-based measures for biclustering of high-dimensional expression data

**DOI:** 10.1186/s13040-018-0165-9

**Published:** 2018-03-27

**Authors:** Juan A. Nepomuceno, Alicia Troncoso, Isabel A. Nepomuceno-Chamorro, Jesús S. Aguilar-Ruiz

**Affiliations:** 1 Departamento de Lenguajes y Sistemas Informáticos, Universidad de Sevilla, Avd. Reina Mercedes s/n, Seville, 41012 Spain; 2 Área de Informática, Universidad Pablo de Olavide, Ctra. Utrera km. 1, Seville, 41013 Spain

**Keywords:** Biclustering of gene expression data, Gene pairwise GO measures, Scatter search metaheuristic

## Abstract

**Background:**

Biclustering algorithms search for groups of genes that share the same behavior under a subset of samples in gene expression data. Nowadays, the biological knowledge available in public repositories can be used to drive these algorithms to find biclusters composed of groups of genes functionally coherent. On the other hand, a distance among genes can be defined according to their information stored in Gene Ontology (GO). Gene pairwise GO semantic similarity measures report a value for each pair of genes which establishes their functional similarity. A scatter search-based algorithm that optimizes a merit function that integrates GO information is studied in this paper. This merit function uses a term that addresses the information through a GO measure.

**Results:**

The effect of two possible different gene pairwise GO measures on the performance of the algorithm is analyzed. Firstly, three well known yeast datasets with approximately one thousand of genes are studied. Secondly, a group of human datasets related to clinical data of cancer is also explored by the algorithm. Most of these data are high-dimensional datasets composed of a huge number of genes. The resultant biclusters reveal groups of genes linked by a same functionality when the search procedure is driven by one of the proposed GO measures. Furthermore, a qualitative biological study of a group of biclusters show their relevance from a cancer disease perspective.

**Conclusions:**

It can be concluded that the integration of biological information improves the performance of the biclustering process. The two different GO measures studied show an improvement in the results obtained for the yeast dataset. However, if datasets are composed of a huge number of genes, only one of them really improves the algorithm performance. This second case constitutes a clear option to explore interesting datasets from a clinical point of view.

## Introduction

Gene expression datasets show the expression profiles of thousand of genes under dozens of samples or experimental conditions. The nature of data motivates a new perspective of clustering where the goal is to discover groups of genes that share the same behavior under a subset of samples and not all of them. These groups of genes with similar profiles under a subset of conditions are called biclusters. Biclustering is a type of clustering where instances (genes) and features (conditions) are simultaneously clustered. Although biclustering algorithms were firstly studied in a general framework with names such as subspace clustering or co-clustering, most of them have been developed in the context of gene expression data [1].

Gene Ontology (GO) is a public repository that stores biological information through a vocabulary of terms [2]. GO has a tree structure composed of three domains or roots: Biological Process (BP), Molecular Functions (MF) and Cellular Components (CC). Each term in this ontology has a set of annotated genes. Terms in higher levels in the tree are more general, while terms in lower levels are more specific and descriptives. Therefore, each gene is related to a set of GO terms with different levels of specificity. Functional annotation files relate a gene to a set of GO terms. GO is usually used in the biclustering field to provide a biological meaning to the results achieved by any biclustering technique [3]. Additionally, the standard framework of comparison among biclustering algorithms is also based on the information stored in GO [4]. All this biological information is used for validation tasks but not for introducing new search criteria in biclustering algorithms. However, nowadays the integration of biological information is one of the challenges and research directions [5]. Knowledge-driven search criteria can be defined by combining co-expression and functional similarity among genes.

Functional similarity measures based on GO establish distances among GO terms. Basically there are two groups of GO measures: graph-based measures and information content (IC)-based measures [6]. The first group is based on the frequency of a term in the GO graph. The second group of measures assumes that the specificity of a term can be directly inferred from its depth in the GO graph. Due to each gene is associated with a set of GO terms, a distance between two genes can be defined according to their information stored in GO. Similarity measures that simultaneously compare sets of terms rather than single terms are more efficient to be used as a measure among genes. These measures are usually called gene pairwise GO measures.

Gene pairwise GO measures are used in this paper in a scatter search-based biclustering algorithm as part of its search criteria. Thus, GO information adds a bias during the search process that improves the algorithm performance. Hence, those biclusters composed of functionally coherent genes are emphasized. The proposed algorithm follows the same search procedure that the algorithm presented in [7]. Each bicluster is sequentially found through a scatter search procedure. This procedure optimizes a fitness function, which involves the gene expression data along with the GO annotation information by means of a gene pairwise GO measure. Therefore, several fitness functions can be defined according to the gene pairwise GO measures to be used. The scatter search is a population-based evolutionary optimization method that emphasizes systematic processes against random procedures. The optimization process is based on the evolution of a small set of solutions that is built with a group of solutions selected by considering intensification and diversity strategies for each iteration. Scatter search carries out a number of fitness function evaluations during the search less than other evolutionary metaheuristics [8]. This paper follows the preliminary ideas presented in [9] and it extends the work previously published in [10], where a particular gene parwise GO measure was studied in the context of biclustering. The impact of biological information integration in the context of high-dimensional datasets is analyzed for the first time in this paper to the best of our knowledge.

The rest of the paper is organized as follows. A short survey of biclustering and some related works are presented in “Related work” section. “Method” section presents the proposed algorithm. Firstly the fitness function and two different options to integrate the biological information by means of two gene pairwise GO measures are provided (“Fitness function” and “Gene pairwise GO measures” subsections). Secondly, the main ideas of a scatter search along with the pseudocode of the algorithm are explained (“Scatter search based-scheme” section). Experimental results and discussion are shown in “Experiments” section. This section also includes a biological study of several biclusters in order to show their relevance in “Biological study” subsection. Finally, conclusions and future works are presented in “Conclusions” section.

## Related work

The main idea of biclustering is to discover local patterns rather than global patterns in datasets. In the last years, many biclustering algorithms have been proposed in the context of gene expression data [11, 12]. These algorithms differ depending on their search criteria and their heuristic strategies [1]. They can be classified according to whether they are based or not on a particular evaluation measure [13]. It is important to note that the comparison among this kind of techniques is a hard task because the best algorithm generally depends on the type of patterns to discover and the nature of the studied dataset [14].

Several algorithms that are usually referenced can be highlighted. They can be considered as classic biclustering algorithms [15]. Cheng and Church [16] and FLOC algorithms [17] find biclusters with a score under a threshold called *Mean Square Residue* (MSR). The first one was the foundational algorithm and the FLOC improved it. Although the MSR measure has been used in many measure-based algorithms, it can not capture some relevant patterns [18]. xMotifs algorithm [19] iteratively searches conserved gene expression subsets of genes that are simultaneously conserved across a subset of conditions. Binary inclusion-maximal biclustering algorithm (BIMAX) was presented in [20] where it was used as a reference method for comparison with other algorithms. The Plaid Model [21] is an additive biclustering algorithm based on additive layers to capture biclusters. Spectral Biclustering [22] uses a checkboard structure to find biclusters and it applies a singular value decomposition (SVD) of the matrix representing the dataset. Factor analysis for bicluster acquisition (FABIA) [23] is based on a statistical method, which studies the variability among variables (genes) according to a potentially lower number of unobserved variables called factors. Order-preserving submatrix algorithm (OPSM) [24] sequentially searches for biclusters based on a linear ordering among rows. Iterative signature algorithm (ISA) [20] finds up-regulated and down-regulated patterns using a nondeterministic greedy search as heuristic. Blocks of coherent values with respect to rows and columns are found by reordering the input matrix. Finally, it can be also highlighted a family of measure-based algorithms that use evolutionary computation techniques such as [25–28]. Moreover, it can be noted that several algorithms of this group use correlations among genes as a measure for purposes of bicluster evaluation [7, 29–35].

In the last years, the use of biological information as a mechanism of knowledge-driven search has been studied. Concretely, some algorithms recently used GO functional files to improve their performance in traditional clustering of gene expression data [36]. GO was also used in an unsupervised scenario based on a Principal Component Analysis (PCA) method in order to explore gene expression datasets [37].

In the field of biclustering, the AID-ISA algorithm [38] is a modified version of the ISA algorithm that uses a procedure to incorporate additional sources of information. GenMiner [39] is an algorithm based on association rules that also handles biological annotation files. It integrates gene expression and annotation data in a single framework in order to select relevant rules during the search process. The algorithm presented in [40] works with self-organizing maps and combines an ontology-based clustering using GO and an expression-based clustering. Moreover, in this field but specialized in microRNA and target genes data, the algorithm presented in [41] used GO in order to establish a ranking from its results.

Due to the NP-hard nature of biclustering [42], most of algorithms have difficulties to find relevant information with high-dimensional datasets. Recently, some authors have included some constrains during the search process in order to deal with the size of the dataset. Thus, only the most relevant part of the dataset is explored [43, 44]. The BiC2PAM algorithm [45] uses pattern mining-based ideas to prune the search process. It also considers the biological context through the fulfilment of several constraints related to interesting properties from a biological point of view and to annotations from domain knowledge. This paper also establishes a classification of the new biclustering algorithms based on knowledge integration: constraints with *nice* properties, parametric constraints and biclustering with annotations.

The authors of this paper presented a preliminary biclustering algorithm that integrates biological knowledge in [9]. Namely, a scatter search metaheuristic algorithm [46] was adapted to optimize a merit function that handled gene expression and gene annotation data. As a consequence of this first study of biological information integration in biclustering, a gene pairwise GO-measure was also studied in [10]. The current work constitutes an extension of this last work in order to analyze how to improve the algorithm performance using these ideas in the context of high-dimensional gene expression datasets. This work can be classified as a constraint-based biclustering algorithm with knowledge integration through the use of annotations from knowledge-based repositories [45].

## Method

The proposed algorithm integrates biological information to search biclusters in gene expression data. A fitness function that characterizes biclusters is defined and it is optimized by means of a scatter search metaheuristic. Basically, two ideas can be differentiated. Firstly, the fitness function definition where a term deals with a functional annotation file to integrate the biological information. Secondly, a search procedure based on a scatter search metaheuristic that minimizes this function. Therefore, the search scheme and the search criterion are independent.

The input data of the algorithm are the gene expression data matrix, the gene functional annotation information and the number of biclusters to find. Each row in the gene expression data matrix is the expression profile of a gene and each column is a sample or experimental condition. Hence, each numerical value of the matrix is the expression value of a gene under a specific condition. Gene functional annotation files relate genes to a set of terms where they are annotated. In this work, GO annotation files are used, being related each gene to a set of GO terms.

### Fitness function

The minimization of the fitness function provides the resultant biclusters. Three different criteria are considered: the volume, the patterns to find in the gene expression matrix and the biological information of the set of genes from GO. Given a bicluster composed of *N* genes and *Q* conditions, the fitness function is defined as follows:

1 $$ f(B) = M_{1}\cdot \frac{1}{N\cdot{Q}}+M_{2}\cdot f_{corr}(B)+M_{3}\cdot f_{GO}(B)  $$

where the first term measures the volume, the second term uses the average correlation to find shifting and scaling patterns and the third one the GO information. *M*_1_, *M*_2_ and *M*_3_ are parameters to weight the relevance of these three terms, respectively.

The average correlation to find particular patterns such as shifting and scaling patterns has been previously used [7]. This term is based on the correlation by pairs of genes and it is defined as:

2 $$ f_{corr}(B) = 1-\frac{1}{\binom{N}{2}}\sum_{i=1}^{N-1}\sum_{j=i+1}^{N}|\rho_{ij}|  $$

where *ρ*_*ij*_ is the pearson correlation coefficient between the genes *g*_*i*_ and *g*_*j*_. Note that this correlation is calculated using the rows and the columns of the submatrix that contain the bicluster information from the gene expression matrix. Due to the best value for the correlation is equal to 1, and the goal is to minimize the fitness function, *f*_*corr*_ is modified to achieve its optimal value when the average correlation is set to 0. The absolute value is considered to capture positive and negative correlations.

The third term in the fitness function handles the GO information of the set of genes in bicluster. The idea is to measure the functional similarities among genes using a gene pairwise GO measure. This term is defined as follows:

3 $$ f_{GO}(B) = 1-\frac{1}{\binom{N}{2}}\sum_{i=1}^{N-1}\sum_{j=i+1}^{N} GOmeasure(g_{i},g_{j})  $$

where *GOmeasure*(*g*_*i*_,*g*_*j*_) represents the value of a determined GO measure for the genes *g*_*i*_ and *g*_*j*_. As it is the case for the previous term mentioned, this term is modified to achieve the optimal value when the average of the GO measure is set to 0. Note that this term can be configured depending on the GO measure to be selected.

Thus, the first and the second terms in the fitness function use the gene expression matrix while the third term uses the gene functional annotation file. The parameters *M*_1_, *M*_2_ and *M*_3_ control the relevance of each term.

### Gene pairwise GO measures

Gene pairwise GO measures provide a distance between two genes according to their corresponding GO terms. These GO-based measures are based on the comparison of a set of terms simultaneously in spite of studying separately single terms. This information is extracted from the gene functional annotation file used as input. These files are built such that for each gene the extended set of its annotations, which includes a direct annotation and their ancestral terms up to the root node, are considered. Two gene pairwise GO measures deeply studied in [6] have been selected to use as the third term of the fitness function (Eq. 1).

#### SimUI measure

This measure is based on counting terms in the graph of GO [6]. It also uses an extra file with the GO structure along with the gene annotation file. Given two genes *g*_1_ and *g*_2_, the *simUI* measure is defined as follows:

4 $$ SimUI(g_{1},g_{2})=\frac{{COUNT}_{t\in{GO(g_{1})\cap GO(g_{2})}}}{{COUNT}_{t\in{GO(g_{1})\cup GO(g_{2})}}}  $$

where *COUNT* is a function to count the number of GO terms.

#### SimGIC measure

This measure is an IC measure that calculates the probability of each term in GO. In addition to the gene annotation file, this measure uses as input an extra file with the GO structure to compute the IC. It shows the best performance when compared to other measures in the experimental study presented in [6]. Given two genes *g*_1_ and *g*_2_, the *simGIC* measure is defined as follows:

5 $$ SimGIC(g_{1},g_{2})=\frac{\sum_{t\in{GO(g_{1})\cap GO(g_{2})}}IC(t)}{\sum_{t\in{GO(g_{1})\cup GO(g_{2})}}IC(t)}  $$

where *IC*(*t*_*i*_)=−*log*(*p*(*t*_*i*_)) is the information content of the term i and *p*(*t*_*i*_) the probability of a term occurring in the corpus. This probability *p*(*t*_*i*_) can be calculated as:

6 $$ p(t_{i})=\frac{frec(t_{i})}{frec(root)}  $$

where:

- *frec*(*root*) is the number of times that a gene is annotated with any term within the ontology.
- $frec(t_{i})=|annot(i)|+\sum _{c\in {children(t_{i})}}|annot(i)|$, where *children*(*t*_*i*_) is the set of all children terms for the term *t*_*i*_ and |*annot*(*i*)| is the number of times being the term annotated.

Note that the graph structure of GO is necessary to compute *children*(*t*_*i*_).

### Scatter search based-scheme

The proposed algorithm is based on a scatter search metaheuristic that optimizes the fitness function (Eq. 1). It follows the same ideas that the algorithm proposed in [9], which uses the search engine of the algorithm published in [46]. Each bicluster is found sequentially through the scatter search procedure that is repeated until the number of biclusters to discover is achieved. Therefore, every search is independent of the previous one. Scatter search is a population-based metaheuristic that generates solutions, which represent biclusters, and the resultant bicluster is the best solution found by the search process.

Biclusters are encoded as two binary strings where the bits indicate their corresponding gene or condition in the gene expression matrix. The main concepts are the intensification of solutions in order to find the optimum and the diversification in order to avoid local minima. As it can be seen in the the scatter search procedure (Algorithm 1), both intensification and diversification strategies are reached through the evolution of a small set of solution called *reference set*.



An *initial population* is generated by the *diversification generation method* with solutions as scatter as possible. These solutions are built from a seed solution following a diversity rule for binary strings [46]. The Hamming distance is used to measure the distance among them. Then, the solutions in the initial population are improved by the *improvement method* (lines 1 and 2 in Algorithm 1). This improvement method is a local search that intensifies the process because each solution is swapped by another solution with a lower value for the fitness function. New solutions are generated using bits permutation in order to be close of the original solution. If none of them improves the original, it remains in the search process [9]. It is important to note that this improvement method is a blind search, and therefore, it is independent of the semantic of the fitness function.

The reference set is built with the most representative solutions from the initial population according to quality and diversity criteria. The five best solutions from fitness function point of view and the five most scattered solutions to these ones are chosen (line 3 in Algorithm 1). The initial population is updated by removing these ten solutions (line 4 in Algorithm 1).

The reference set evolves until it is stable, namely, until every new solution is worst than the solutions stored in the reference set (line 7 to 12 in Algorithm 1). The *subset generation method* generates new binary strings giving rise to new solutions when applying the *solution combination method*. This method is based on traditional crossover operators normally used with binary strings. The *reference set update method* consists in choosing the 10 best solutions from the new generated solutions and the solutions that form the reference set. The reference set is rebuilt and the previous process is repeated a number of times (line 13 and 14 in Algorithm 1). The output is the best bicluster in the last reference set.

Some inner parameter values are required by the algorithm such as the number of solutions of the initial population, the size of the reference set and the maximum number of iterations of the scatter search. They are 200, 10 and 20, respectively and they have been chosen according to the scatter search literature [8]. It must be noted that the algorithm does not need to control the size of other inner generated subsets of solutions.

## Experiments

The experiments have been designed to study the effect of using biological information by means of gene pairwise GO measures on biclustering. The goal is to determine which is the best measure and its best parameter configuration to use in the context of high-dimensional gene expression datasets. Therefore, the purpose is to compare the effect of each measure on the biclustering process more than to study the search procedure itself. The biclusters obtained by different fitness functions based on the two GO measures detailed in “Gene pairwise GO measures” section have been analyzed. In addition, three possibilities for the parameter configuration have been considered for each fitness function. Finally, the biclusters provided when no considering the biological information integration through a particular fitness function configuration are also analyzed.

The input data of the algorithm are the gene expression matrix along with a gene annotation file. These annotation files link each gene with a set of GO terms. The gene functional annotation file is a gene association file with the extension.*goa* downloaded from Gene Ontology (GO). An extra file with the extension.*obo* in order to provide extra information for each GO term has also been downloaded. The genes nomenclature must be the same in the annotation file and in the expression matrix file. Genes must share the same identifiers in both files in order to be able to connect them. Hence, it is recommended to use standard gene names in the expression matrix.

The sets of biclusters obtained by the scatter search for each run have been studied in accordance with their percentage of enriched biclusters. This criterion is commonly used to establish a comparison among biclustering algorithms and their performance for biological data [3, 4]. Thus, a ranking of different sets of biclusters can be made depending on the percentages of enrichment. The detection of statistically overrepresented GO terms has been done with the hypergeometric test [47], multiple-testing adjustments with the Benjamini and Hochberg false discovery rate [48] with a significance level of *α*=0.05. The results reported here have been carried out using the Biological Process (BP) domain of GO.

These experiments have been firstly focused on three yeast datasets previously used in [10] and in [49]. These first cases constitute an example of standard size datasets in biclustering literature. Secondly, a group of human datasets related to clinical data of cancer have been used in the experimental study. These data are composed of a huge number of genes and several of them can be considered examples of high-dimensional gene expression datasets [15].

### Data sets description

Three *Saccharomyces cerevisiae* datasets and several *Homo sapiens* datasets from *NCBI Gene Expression Omnibus* repository [50] have been used in these experiments. The first dataset is composed of 882 genes and 131 samples after being preprocessed. The identifier of this yeast dataset is *GDS*1116 in the repository. The other two yeast datasets have been downloaded from the supplementary information provided in [49]. They have been labeled as 15*mM*_*diamide* and 25*mM*_*DTT* and their size is (996×8) and (1025×8), respectively. The raw data of the human datasets were generated in the context of clinical experiments with patients that suffer different cancer diseases. Table 1 shows information for each human dataset and the clinical study where they were generated in addition to the accession numbers or identifiers in the repository and sizes after being preprocessed. It is important to emphasize the huge number of genes for most of them. For this reason, most of them can been considered high-dimensional gene expression datasets from a biclustering perspective.

**Table 1:** Human datasets related to cancer clinical data used in the experimental study

Dataset	Size	Information about the experimental context of data
GDS3289	(971 ×104)	A prostate cancer study of the disease progression from beginning epithelium
		to metastatic stage.
GDS2415	(1690 ×59)	A breast carcinoma tumor study in patients with breast-conserving therapy.
GDS2918	(4587 ×20)	A study of blood plasma from patients with colorectal cancer.
GDS3966	(10296 ×83)	An analysis of melanoma samples in different stages of the disease.
GDS3139	(12270 ×29)	A histological analysis of normal breast epithelia in patients with breast cancer.
GDS4794	(16925 ×65)	A lung cancer study of small cells in initial stages of the disease.

Raw data have been preprocessed using Babelomics web tool [51]. Missing values have been replaced by the mean of the values for each row (gene). The rows with a percentage of missing values greater than 30% have been removed. If a gene appears several times in the raw data these rows (genes) have been summed up by means of the median of the values. The 15*mM*_*diamide* and 25*mM*_*DTT* yeast datasets were previously processed but their gene nomenclature was different from the.*goa* yeast annotation file. Therefore, their gene names have been translated from *ORFF format* to standard gene names using the *YEASTRACT* [52].

### Results

Two different definitions for the fitness function are possible depending on the gene pairwise GO measure to be used to integrate biological information in the bicluster search process. The third term (Eq. 3) in the fitness function can be based on the simUI or the simGIC measures. On the other hand, this term is null if there is not any kind of biological integration. For each gene pairwise GO measure, three parameter configurations are studied, (211), (212) and (221), where these numbers are the values for *M*_1_, *M*_2_ and *M*_3_ in Eq. 1, respectively. The first configuration for the fitness function, (211), provides the same relevance to the average correlation (Eq. 2) as to the GO measure (Eq. 3). The second configuration, (212), emphasizes the GO measure over the average correlation. Finally, the average correlation is more relevant when using the (221) configuration. The parameter *M*_3_ is set to 0 when the biological information integration is not taken into account, and hence, there is only two possible configurations, (210) and (220), in this case. It is important to highlight that the three terms in the fitness function vary between 0 to 1. On the other hand, the parameter *M*_1_ is set to 2 for all configurations due to the previous experience shows that the volume must be equal or more relevant that the average correlation in order to avoid trivial biclusters [9]. Therefore, a total of eight possibilities are studied in order to run the scatter search algorithm for each dataset, that is, two GO measures with three possible parameters configurations and two possible configurations without considering biological information. Note that *M*_1_ and *M*_2_ can not be equal to zero in order to avoid trivial biclusters and to control the number of conditions during the search, respectively.

**Fig. 1:**
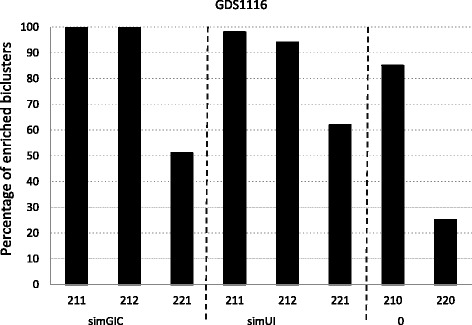
Biclusters obtained by the biclustering algorithm for each fitness function for the GDS1116 yeast dataset

**Fig. 2:**
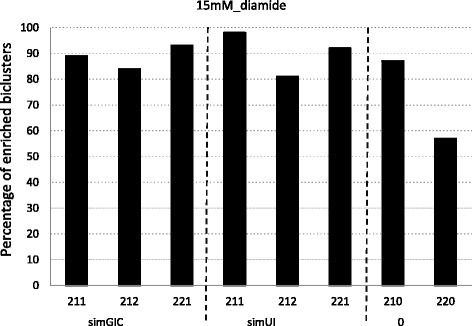
Biclusters obtained by the biclustering algorithm for each fitness function for the 15*mM*_*diamide* yeast dataset

**Fig. 3:**
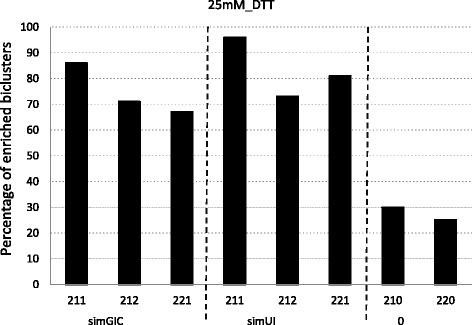
Biclusters obtained by the biclustering algorithm for each fitness function for the 25*mM*_*DTT* yeast dataset

Figures 1, 2 and 3 report the percentage of enriched biclusters obtained by the scatter search algorithm for all configurations and for *GDS*1116, 15*mM*_*diamide* and 25*mM*_*DTT* yeast datasets, respectively. Namely, biclusters provided for the (211), (212) and (221) configurations for the simGIC and the simUI measures and for the (210) and (220) configurations where no biological information integration is present are compared. A number of 100 biclusters have been obtained for all runs in order to have a wide range of results to handle the random nature of the algorithm. It should be noted that although the complete information of GO is used during the search, it is only used the Biological Process (BP) sub-ontology in order to do this enrichment study.

Likewise, Table 2 presents the information about biclusters obtained from the application of the scatter search to human datasets such as *GDS*3289, *GDS*2415, *GDS*2918, *GDS*3966, *GDS*3139 and *GDS*4794. The second and the third column show the different fitness function definitions and possible parameter configurations, respectively. Concretely, the simUI and the simGIC measures with (211), (212) and (221) configurations or no measure for biological integration corresponding to (210) and (220) configurations. The fourth column reports the average size of the set of biclusters for each run, namely, the average number of genes and conditions. Finally, the percentage of enriched biclusters is shown in the fifth column.

**Table 2:** Biclusters obtained by the biclustering algorithm for each fitness functions for *GDS*3289, *GDS*2415, *GDS*2918, *GDS*3966, *GDS*3139,*GDS*4794 datasets

	Fitness function			Enriched
Dataset		Parameters	Size	biclusters (%)
	GOmeasure	(*M*_1_, *M*_2_, *M*_3_)		BP
		(2, 1, 1)	(15.3 ×14.0)	38
	simUI	(2, 1, 2)	(9.4 ×14.0)	57
		(2, 2, 1)	(20.0 ×3.0)	39
		(2, 1, 1)	(17.1 ×13.5)	28
GDS3289	simGIC	**(2, 1, 2)**	(10.6 ×14.0)	**92**
		(2, 2, 1)	(28.4 ×3.0)	18
		(2, 1, 0)	(21.6 ×14.3)	1
	0	(2, 2, 0)	(40.6 ×3.4)	5
		(2, 1, 1)	(18.1 ×13.9)	4
	simUI	(2, 1, 2)	(9.1 ×13.4)	36
		(2, 2, 1)	(21.9 ×3.1)	8
		(2, 1, 1)	(22.9 ×3.1)	0
GDS2415	simGIC	**(2, 1, 2)**	(13.4 ×12.5)	**44**
		(2, 2, 1)	(35.3 ×3.0)	8
		(2, 1, 0)	(23.2 ×13.4)	0
	0	(2, 2, 0)	(41.7 ×3.2)	0
		(2, 1, 1)	(40.3 ×6.8)	1
	simUI	(2, 1, 2)	(40.0 ×6.8)	0
		(2, 2, 1)	(53.3 ×3.4)	2
		(2, 1, 1)	(31.0 ×6.7)	10
GDS2918	simGIC	**(2, 1, 2)**	(13.3 ×7.3)	**42**
		(2, 2, 1)	(34.4 ×3.4)	23
		(2, 1, 0)	(36.5 ×7.2)	1
	0	(2, 2, 0)	(54.0 ×3.3)	1
		(2, 1, 1)	(26.2 ×17.8)	4
	simUI	(2, 1, 2)	(26.5 ×18.3)	6
		(2, 2, 1)	(41.8 ×3.4)	1
		(2, 1, 1)	(23.2 ×17.8)	11
GDS3966	simGIC	**(2, 1, 2)**	(13.9 ×19.1)	**45**
		(2, 2, 1)	(35.7 ×3.3)	6
		(2, 1, 0)	(26.5 ×18.3)	3
	0	(2, 2, 0)	(42.3 ×3.4)	0
		(2, 1, 1)	(35.6 ×13.0)	2
	simUI	(2, 1, 2)	(35.2 ×13.2)	3
		(2, 2, 1)	(28.9 ×9.7)	1
		(2, 1, 1)	(31.6 ×13.4)	7
GDS3139	simGIC	**(2, 1, 2)**	(18.4 ×13.1)	**23**
		(2, 2, 1)	(26.9 ×9.9)	4
		(2, 1, 0)	(35.1 ×13.4)	2
	0	(2, 2, 0)	(27.7 ×9.8)	3
		(2, 1, 1)	(26.0 ×17.1)	5
	simUI	(2, 1, 2)	(25.7 ×17.2)	3
		(2, 2, 1)	(46.3 ×3.8)	0
		(2, 1, 1)	(23.7 ×16.7)	13
GDS4794	simGIC	**(2, 1, 2)**	(17.0 ×16.43)	**28**
		(2, 2, 1)	(36.6 ×3.7)	10
		(2, 1, 0)	(25.7 ×17.1)	4
	0	(2, 2, 0)	(46.8 ×3.8)	1

**Fig. 4:**
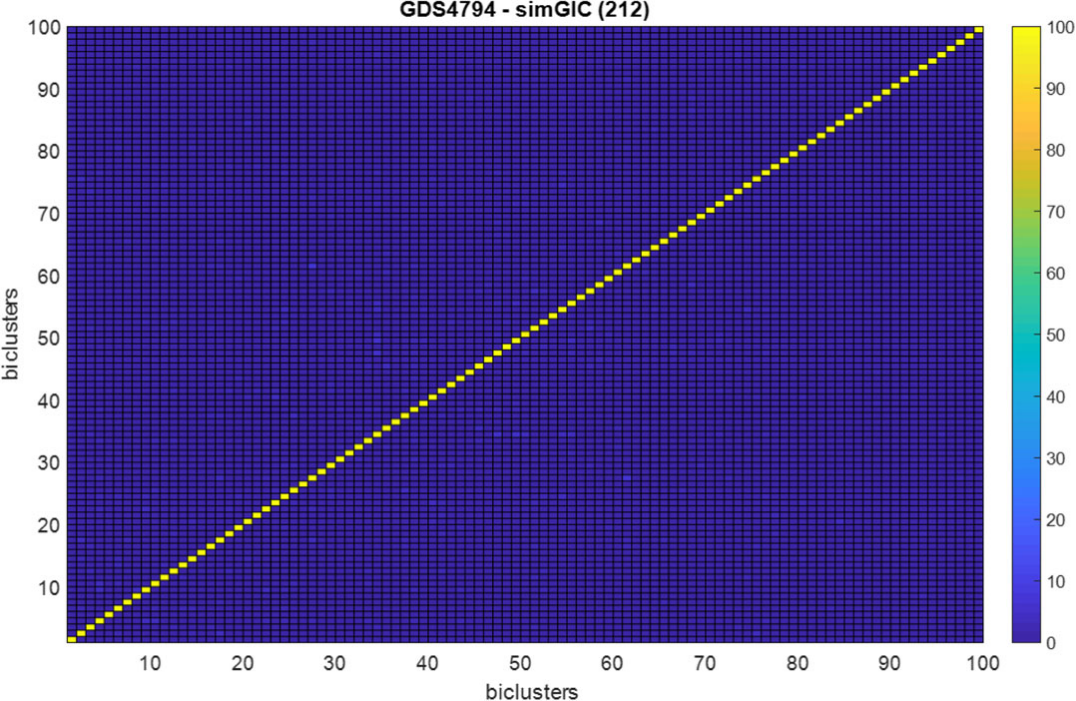
Overlapping among biclusters obtained by (212)-simGIC fitness function configuration for GDS4794 dataset

**Fig. 5:**
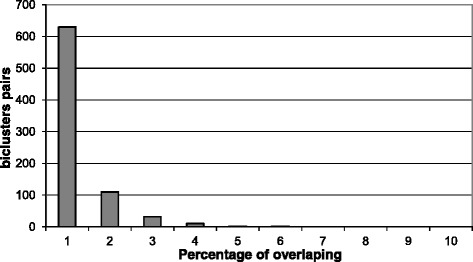
Histogram of percentage of overlapping among biclusters obtained by (212)-simGIC fitness function configuration for GDS4794 dataset

**Fig. 6:**
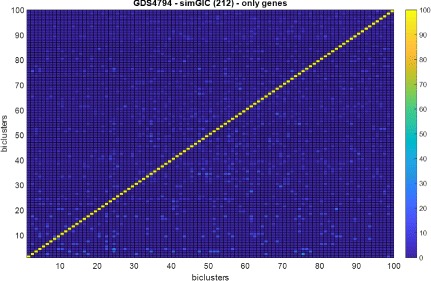
Overlapping among biclusters obtained by (212)-simGIC fitness function configuration for GDS4794 dataset studying only their genes

The Figs. 4 and 5 show the overlapping among 100 biclusters obtained by the fitness function based on simGIC measure with the (212) setting for *GDS*5794. Each element in the matrix of the heatmap is the percentage of overlapping between two biclusters. It can be observed a low overlapping among the obtained biclusters. The reason is that each bicluster is found by independent scatter search processes that uses a different initial population [53]. Therefore, it is not necessary to introduce a control of overlapping in the algorithm in special in the context of high-dimensional datasets. Note that all biclusters are overlapped with a percentage below 10%. Additionally, Fig. 6 show the overlapping but considering only the set of genes in each bicluster. It can also be observed a low overlapping.

### Discussion of the results

Figure 1 studies a case composed of 882 genes and 131 conditions. The best parameter setting is provided when the *M*_2_ parameter is equal to 1. From the Fig. 1, it can be observed that a less percentage of enriched biclusters is obtained when using the (221) configuration than that of (211) or (212) configurations for the two fitness functions. Furthermore, it can also be observed that without biological information integration the (210) configuration obtains better biclusters regarding the enrichement than the (220) configuration. Therefore, configurations where the GO measure has more relevance or the same as the average correlation improve the algorithm performance. Moreover, the integration of biological information clearly improves the quality of the biclusters. It can be appreciated that the biclusters for simGIC and simUI measures are better than that obtained without any biological information. Finally, it can be observed that both gene pairwise GO measures show similar results, highlighting the biclusters provided when applying simGIC and simUI measures for (211) and (212) configurations with more than a 90% of enriched biclusters.

Figures 2 and 3 study datasets with sizes (996×8) and (1025×8) respectively. Note that although they have approximately a similar number of genes to the previous dataset, *GDS*116, they only have 8 conditions. It can also be observed a similar behavior in Fig. 1. The integration of biological information improves the performance of the algorithm specially in Fig. 3. Moreover, both measures show a similar behavior although simUI presents slightly better results than simGIC.

Table 2 presents a group of experiments for datasets with a very large number of genes, concretely, 971, 1690, 4587, 10296, 12270 and 16925, respectively (see Table 1). Note that from this table, the datasets are in ascending order regarding the number of genes. For these high-dimensional datasets, it can be firstly observed that the simGIC measure introduces a bias during the search process, and as a consequence, the scatter search algorithm improves giving rise to better biclusters. In this context, the biclustering algorithm has problems to find enriched biclusters but the simGIC measure clearly makes the search process more effective. From this table, it can also be observed that the (212) configuration shows higher enriched bicluster percentages than the rest of configurations for the simGIC measure. In particular, values marked in bold reveal a 92%, 44%, 42%, 45%, 23% and 28% of enriched biclusters for *GDS*3289, *GDS*2415, *GDS*2918, *GDS*3966, *GDS*3139 and *GDS*4794 datasets, respectively. On the other hand, the simUI measure improves the performance of the biclustering algorithm for *GDS*3289 and *GDS*2415 datasets when used the 212 configuration finding a 57 and 36% of enriched biclusters. However, this behavior changes when the number of genes in the dataset increases considerably. It should be appreciated that all datasets are formed by a number of genes much greater than the number of genes in *GDS*3289 and *GDS*2415 datasets. As it was expected, the higher the number of genes, the lower percentage of enriched biclusters is.

In summary, these experiments show that the integration of biological information by means of the two GO measures proposed here improves the scatter search algorithm performance when using datasets of small o moderate size, showing similar results for both measures. However, if the dataset is composed of a huge number of genes, the biological integration must be defined using the simGIC measure and the (212) parameter configuration.

### Biological study

The resultant biclusters obtained by the (212)-simGIC fitness function definition for the GDS4794 dataset have been biologically studied. This dataset is a high-dimensional dataset related to lung cancer. Due to its huge number of genes, this dataset is supposed to be the most difficult to explore by the biclustering algorithm. This biological study has been focused on the subset of the 28 enriched biclusters (see Table 2). Table 3 shows that 24 biclusters out of 28 contain genes associated with cancer diseases. This table has been built matching the list of oncogenes, candidate cancer genes provided by the *Network of Cancer Genes* (NCG) [54] and the genes in each bicluster jointly.

**Table 3:** Group of enriched biclusters related to cancer obtained with the (212)-simGIC fitness function for the GDS4794 dataset

id.	Oncogenes	Candidate cancer genes	Number of
biclusters			genes in
			each bicluster
*bi*_2	BRIP1		6
*bi*_13	CRTC1, KLF6	ERF	8
*bi*_19	PIK3R1		12
*bi*_21	FANCD2		11
*bi*_22	SMARCE1		13
*bi*_32	ATP2B3	AMPH, ANK2	18
*bi*_41	RPL22		12
*bi*_53	BLM, MSH2, REL, MYC	SMAD2	18
*bi*_63	EZH2, TFE3, ACSL6		28
*bi*_65	ELF4	GNA13	11
*bi*_82	PALB2, TPR, NUP98, NUP214	CHD4, DBR1	16
*bi*_4		ZHX2	7
*bi*_11		CLCN4	9
*bi*_15		SNRPA, DBR1	13
*bi*_25		NR3C2, CHD2	18
*bi*_26		TTK, PHIP, GLI3	16
*bi*_29		GRM3	10
*bi*_34		PRKCG, RASGEF1A	17
*bi*_35		CACNA2D1	16
*bi*_39		PTPRT, NGEF, GRIA3, CHST1, DUSP7	17
*bi*_70		AMPH, BRINP3, SPTBN4, RBMX	22
*bi*_72		NCOR1, NCOR2, RBMX, TCEB1	19
*bi*_89		PPM1D, TDG, RNF103, CTIF	17
*bi*_100		SLC25A48, TAF1, RASSF6	46

The hypothesis is that the algorithm can detect biclusters functionally coherent. Therefore, these biclusters that contain cancer genes should be functionally related with some biological processes of cancer. In order to determine the potential biomedical relevance of these biclusters, they have been analyzed using *FuncAssociate* [47] and their reported GO terms have been studied from a cancer perspective using the *Integrated human lung cancer-related factors database* (IHLDB) [55]. Besides, *Reactome* [56] has also used as a resource for mapping genes in signalling pathways.

**Fig. 7:**
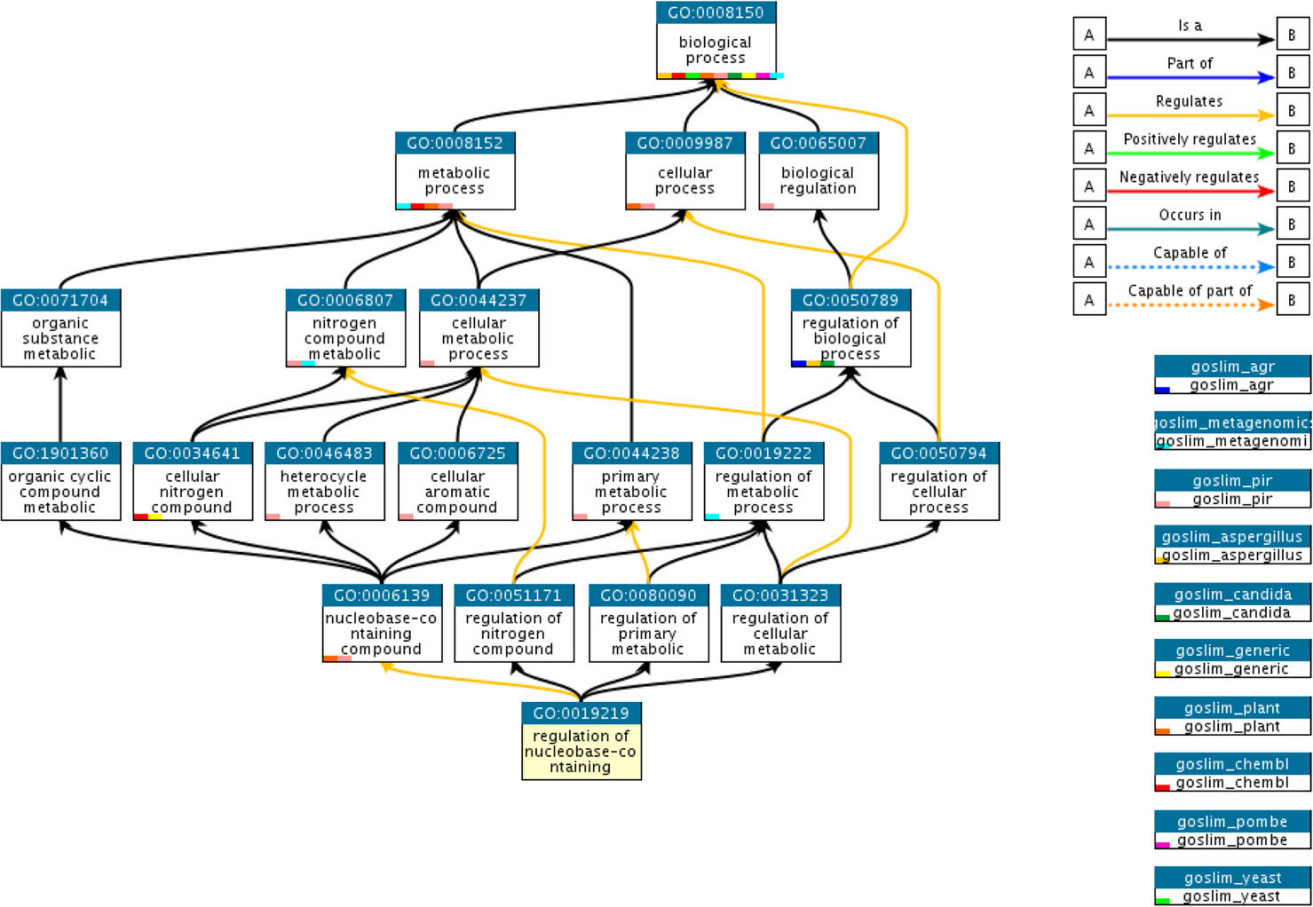
Biological study of biclusters obtained by the (212)-simGIC configuration for the GDS4794 dataset: highlighted GO term observed in the results for GDS4794 dataset

Firstly, the study has been focused on the bicluster labeled as *bi*_2 in Table 3. This bicluster contains the *BRIP*1 gene which is a recessive cancer gene mutated in multiple primary sites. The analysis with *FuncAssociate* of its six genes reports several GO terms. It must be highlighted the term GO:0019219 where the six genes are simultaneously annotated. This GO term is not only related to the *BRIP*1 according to the NCG but also it’s a GO term related to lung cancer according to the information provided by IHLDB. This term is linked with the nucleotide and nucleic acid metabolism and it is in the level 6 of GO. Figure 7 shows GO:0019219 inside the Biological Process domain of GO using *QuickGO* [57].

Secondly, *bi*_53, *bi*_63 and *bi*_82 biclusters, with 4, 4 and 3 oncogenes respectively, have also been analyzed using *Reactome*. These three biclusters have in common the pathway named Cell cycle and mitotic. This process, which is responsible of the cell progresses and its division, is the key of cancer diseases [58]. Note that this pathway has a high number of entities, namely, 568. For example, the *bi*_53 is composed of 18 genes and it has 7 genes identified in the pathway. Table 4 shows pathways that have the word cancer in their names reported by *Reactome* for the *bi*_53 bicluster. It can be highlighted the first two with a very low FDR and with 2 of a total of 3 genes matched in the pathway. The complete information about the 133 pathways reported for this bicluster is included as an excel file in the link of Availability of data and materials.

**Table 4:** Mapping analysis provided by Reactome for the *bi*_53_ bicluster obtained with the (212)-simGIC fitness function

Pathway identifier	Pathway name	FDR	Entities	Entities
			found	total
R-HSA-3304347	Loss of Function of SMAD4 in Cancer	5.27E-11	2	3
R-HSA-3311021	SMAD4 MH2 Domain Mutants in Cancer	5.27E-11	2	3
R-HSA-3304356	SMAD2/3 Phosphorylation Motif Mutants in Cancer	0.002	2	7
R-HSA-3304349	Loss of Function of SMAD2/3 in Cancer	0.002	2	9
R-HSA-3315487	SMAD2/3 MH2 Domain Mutants in Cancer	0.002	2	9
R-HSA-3656532	TGFBR1 KD Mutants in Cancer	0.002	2	9
R-HSA-3656534	Loss of Function of TGFBR1 in Cancer	0.002	2	9
R-HSA-3304351	Signaling by TGF-beta Receptor Complex in Cancer	0.002	2	10
R-HSA-2894858	Signaling by NOTCH1 HD+PEST Domain Mutants in Cancer	0.017	2	68
R-HSA-2644602	Signaling by NOTCH1 PEST Domain Mutants in Cancer	0.017	2	68
R-HSA-2644603	Signaling by NOTCH1 in Cancer	0.017	2	68

Those pathways that includes the word cancer in their names are presented in this table. The complete information about the 133 found pathways can be downloaded as an excel file in the link of Availability of data and materials

## Conclusions

A biclustering algorithm based on a scatter search scheme that integrates biological information has been studied in this paper. Each bicluster is sequentially found by the scatter search algorithm through the optimization of a merit function. This function is constituted by three different terms dealing with the information provided by inputs files: the gene expression matrix, and additionally, a gene functional annotation file extracted from GO. The third therm in the fitness function is computed by using a gene pairwise GO measure. Two different GO measures giving rise to several different fitness functions configurations have been analyzed in this work.

Parameter settings have been studied analyzing the most representative situations for each fitness function. Experimental results have shown that the algorithm performance is improved when the biological information is integrated. It can be concluded that the use of GO measures drives the search of the algorithm to biclusters composed of groups of genes functionally coherent. The two possibilities of GO information integration have shown a similar behavior for three yeast datasets with approximately one thousand of genes. However, the simGC measure with the (212) parameter configuration is the only measure that improves the algorithm performance for high-dimensional datasets. Moreover, a biological study of the results obtained by the simGIC measure for the cancer dataset, the most difficult dataset to explore due to its number of genes, reveals interesting biclusters from a disease perspective.
